# Anthrax Postexposure Prophylaxis in Postal Workers, Connecticut, 2001

**DOI:** 10.3201/eid0810.020346

**Published:** 2002-10

**Authors:** Jennifer L. Williams, Stephanie S. Noviello, Kevin S. Griffith, Heather Wurtzel, Jennifer Hamborsky, Joseph F. Perz, Ian T. Williams, James L. Hadler, David L. Swerdlow, Renee Ridzon

**Affiliations:** * Centers for Disease Control and Prevention, Atlanta, Georgia, USA; † Connecticut Department of Public Health, Hartford, Connecticut, USA

**Keywords:** Anthrax, *Bacillus anthracis*, prophylaxis, adverse effects, ciprofloxacin, doxycycline, patient noncompliance, Connecticut

## Abstract

After inhalational anthrax was diagnosed in a Connecticut woman on November 20, 2001, postexposure prophylaxis was recommended for postal workers at the regional mail facility serving the patient’s area. Although environmental testing at the facility yielded negative results, subsequent testing confirmed the presence of *Bacillus anthracis*. We distributed questionnaires to 100 randomly selected postal workers within 20 days of initial prophylaxis. Ninety-four workers obtained antibiotics, 68 of whom started postexposure prophylaxis and 21 discontinued. Postal workers who stopped or never started taking prophylaxis cited as reasons disbelief regarding anthrax exposure, problems with adverse events, and initial reports of negative cultures. Postal workers with adverse events reported predominant symptoms of gastrointestinal distress and headache. The influence of these concerns on adherence suggests that communication about risks of acquiring anthrax, education about adverse events, and careful management of adverse events are essential elements in increasing adherence.

On November 20, 2001, *Bacillus anthracis* was confirmed in blood cultures from a 94-year-old woman in rural Oxford, Connecticut, who was diagnosed with inhalational anthrax and died 1 day later (1,2). No obvious source of exposure to *B. anthracis* was identified. She was the 22nd patient diagnosed with anthrax in the United States in 2001 (3). Before this case, all patients diagnosed with inhalational anthrax had had contact with intentionally contaminated mail delivered through the postal system, with the exception of a patient in New York City (where an investigation was under way). Since the source of transmission was identified as the mail for all but one anthrax case, investigation of area postal facilities began immediately.

 The mail was considered a likely source of contamination for the patient in Connecticut, and postexposure antimicrobial prophylaxis was recommended for postal workers employed in the regional distribution center and local post office serving the patient’s area. At the regional postal distribution center, which operates 24 h a day and employs 1,122 workers, employees work one of three 8-h shifts and process approximately 3 million pieces of mail daily.

The regional processing center contains 29 high-speed sorting machines. In contrast, the local post office, a two-room structure with 48 employees, has no high-speed sorting machines. All mail collected in the local post office is sent to the regional processing center. The post office serves two zip code areas; mail to be delivered to the two zip codes is hand-sorted at the local level by carrier route.

The Connecticut Department of Public Health (CDPH), in consultation with the Centers for Disease Control and Prevention (CDC), recommended postexposure prophylaxis as a precaution to protect the health of the postal workers in these facilities (4). As part of a national distribution center sampling protocol, an independent contractor working for the United States Postal Service (USPS) took environmental samples on November 11, but anthrax spores had not been isolated in the regional distribution center. The decision was made to offer prophylaxis to postal workers pending the results of additional, more focused testing.

 The first of many postexposure prophylaxis clinics was held on November 21, 2001. Postal workers were given an initial 10-day course of ciprofloxacin unless contraindicated (5–7). Nasal swabs were collected from the postal workers at the first clinics to determine if contamination was present in the facilities, rather than to diagnose or define individual exposure (8). *B. anthracis* was not isolated from any of 485 nasal swabs taken from postal workers.

On November 21, 25, and 28 and December 2, increasingly focused environmental sampling was performed of both the regional distribution center and the local post office to determine whether any contaminated mail had passed through the facilities (9). Samples obtained on November 21 and 25 were negative; samples taken on November 28 and December 2 from the four high-speed sorting machines in the regional distribution center were positive. No contamination was identified in the local post office. Based on the positive results, the CDPH recommended that prophylaxis be extended for a full course of 60 days for all postal workers in the regional facility. Facility management conducted a progressive series of town hall meetings to notify postal employees of the test results at the various facilities, as well as results of postal worker nasal swabs. Although contaminated sorting machines were shut down for machine-specific decontamination, the regional distribution facility remained opened.

Antimicrobial testing of the Connecticut patient’s isolates confirmed the sensitivity of this *B. anthracis* strain to both doxycycline and ciprofloxacin. For the continuation phase of prophylaxis, doxycycline was offered as the primary antibiotic unless contraindications existed or the workers specifically requested to continue on ciprofloxacin.

On December 10, 2001, we conducted a survey to evaluate postal workers’ adherence to postexposure prophylaxis and to identify factors influencing their degree of adherence. This article describes the findings of the study.

## Methods

 Of the 1,122 postal workers at the regional distribution center, we randomly selected 100 from the night and day shifts. Five workers declined; five additional workers were randomly selected and agreed to participate (refusal rate 5%). CDC health officials interviewed the group of postal workers using a standardized questionnaire to collect information on demographics, adherence, side effects, and attitudes regarding postexposure prophylaxis and exposure risk. Several characteristics were examined for determinants of starting prophylaxis, including sex, race, and age, as well as whether the postal worker worked on high-speed machinery or obtained an influenza vaccine. For comparison, age was divided into quartiles. The lowest quartile (age <37 years) was compared with the top three quartiles, and the highest quartile (age >52) was compared with the bottom three quartiles. Serious side effects were defined as those causing death, hospitalization, persistent or substantial disability, or birth defects, or requiring intervention to avoid these outcomes (10). We conducted our analysis using SAS software, version 8.2 (SAS Institute, Inc., Cary, NC).

## Results

 Of the 100 postal workers sampled, 66% were men. Mean age was 45 years (range 19–65 years). Ethnicities reported were Caucasian (71%), African-American (23%), Asian/Pacific Islander (4%), and Hispanic (2%). None of the respondents were pregnant. Fifteen employees worked on high-speed sorting machines. Forty-two postal workers reported obtaining an influenza vaccine during the previous 3 months.

Ninety-four of the 100 workers surveyed acquired antibiotics from postexposure prophylaxis clinics sponsored by the USPS; 6 workers did not attend the clinics. Of the 94 workers who acquired prophylaxis, only 68 started the antibiotics to prevent anthrax; therefore, of those surveyed, 32 postal workers did not initiate prophylaxis. Postal workers were given ciprofloxacin at initial prophylaxis clinics unless they reported contraindications. Of the 68 postal workers starting antibiotics, 54 persons started ciprofloxacin, 12 doxycycline, and 2 other antibiotics.

 Characteristics of the persons who started prophylaxis versus those who did not are presented in Table 1. Male postal workers were 1.5 times more likely to start prophylaxis than female postal workers (relative risk [RR] 1.52; 95% confidence interval [CI] 1.1 to 2.2; p<0.01). Persons who reported obtaining an influenza vaccine were more likely to start postexposure prophylaxis (RR 1.26; 95% CI 1.0 to 1.6; p=0.07), although this observation did not reach statistical significance. Working on high-speed sorting machines, race, and age were not predictors of starting prophylaxis.

**Table 1:** Characteristics of postal workers starting postexposure prophylaxis, Connecticut, 2001

Characteristic	No. of postal workers (n=100)	Relative risk	Confidence interval	p value
Male	63	1.52	1.1 to 2.2	0.004
Influenza vaccine	42	1.26	1.0 to 1.6	0.07
High-speed machine	15	0.86	0.6 to 1.3	0.47
African-American	22	0.78	0.5 to 1.1	0.13
Age <37 years	22	0.98	0.7 to 1.4	0.92
Age >52 years	26	0.93	0.7 to 1.3	0.63

 We asked the 32 postal workers who never started postexposure prophylaxis to identify all reasons for declining prophylaxis (Table 2) and to indicate the single most important reason. Nineteen (59%) workers stated that they did not feel they were at personal risk for anthrax. Equal proportions of postal workers (47%) cited negative nasal swabs of workers and concerns about side effects as reasons for not starting prophylaxis. Additional reasons included apprehension about antibiotic resistance, waiting to see if personal exposure had occurred, initial negative environmental samples, and fears that prophylaxis would weaken immune systems. When postal workers were asked to identify the single most important reason for not starting postexposure prophylaxis, 25% of workers reported not personally believing they were at risk for anthrax. An additional 13% cited concerns about side effects as the most important reason for not starting the regimen.

**Table 2:** Reasons for postal workers to decline postexposure prophylaxis regimen, Connecticut, 2001

Response	No. of postal workers (n=32)	%
Not at risk for anthrax	19	59
Concerned about side effects from the antibiotics	15	47
Nasal swabs were negative	15	47
Concerned about antibiotic resistance	14	44
Waiting to see if exposed	12	38
Negative environmental samples	12	38
Concerned about weakening immune system	10	31

## Adherence to Postexposure Prophylaxis

 Adherence to the prophylaxis regimen was examined in the 68 workers who started the prophylaxis. We grouped adherence by an average of how many days the worker reported being able to take antibiotic exactly as prescribed. Thirty-one (46%) postal workers reported taking the prophylaxis regimen correctly every day; 23 (34%) took antibiotics correctly 5–6 days per week; and 10 (15%) of workers took antibiotics correctly <4 days per week. Adherence information was not available for four postal workers. Of those starting postexposure prophylaxis, 37 (54%) persons reported missing doses. The top two reasons workers cited for missing a dose were forgetting to take the antibiotic (32%) and side effects (15%).

### Reasons for Stopping Postexposure Prophylaxis

 Twenty-one (31%) of 68 postal workers had discontinued the prophylaxis regimen at the time of the survey. We asked these workers to identify all reasons for discontinuation (Table 3) and to indicate the single most important reason why they stopped. Over half (52%) of all who discontinued believed they were not at personal risk or did not believe they had been exposed to *B. anthracis.* Nine (43%) cited side effects as a reason for stopping. Additional concerns were the initial negative environmental findings and the negative nasal swabs. When postal workers were asked to identify the single most important reason for discontinuing prophylaxis, 33% of postal workers reported experiencing side effects; 19% cited initial negative environmental samples from the facility; and 19% did not feel personally exposed.

**Table 3:** Reasons for discontinuing postexposure prophylaxis regimen, Connecticut, 2001

Response	No. of postal workers (n=21)	%
Not at risk for anthrax	11	52
Not exposed	11	52
Had side effects from the antibiotic	9	43
Nasal swabs were negative	7	33
Negative environmental samples	6	29

### Side Effects

After susceptibility testing of isolates was confirmed, postal workers were switched to doxycycline by USPS physicians, unless that switch was contraindicated; of 47 workers continuing antibiotics, 43 (91%) were switched to doxycycline during the second round of prophylaxis clinics. Six (13%) workers were switched because of side effects. At the time of the survey, postal workers had taken each medication for approximately the same number of days.

 Equal numbers of postal workers surveyed took at least some ciprofloxacin (n=55) and some doxycycline (n=56). Twenty-three (42%) postal workers experienced side effects while taking ciprofloxacin, with 22% reporting multiple symptoms. Twenty-one (38%) postal workers experienced side effects while taking doxycycline, with 21% reporting multiple symptoms. Overall, 35 (51%) of those who began postexposure prophylaxis experienced symptoms while on antibiotics.

 Of side effects most frequently reported by postal workers for both antibiotics, the most common were gastrointestinal complaints (Table 4). Diarrhea and abdominal pain were reported by 22% of workers on ciprofloxacin and 13% of workers on doxycycline. Nausea and vomiting were reported by 15% of the postal workers taking ciprofloxacin and 18% taking doxycycline. Fatigue was cited by 9% of the postal workers taking either drug. No significant differences between the proportions of postal workers reporting side effects while taking either medication were reported. No serious side effects were noted.

**Table 4:** Side effects reported, by antibiotic, Connecticut, 2001

Side effect reported	Ciprofloxacin (%) (n=55)	Doxycycline (%) (n=56)
Diarrhea/abdominal pain	12 (22)	7 (13)
Nausea/vomiting	8 (15)	10 (18)
Fatigue	5 (9)	5 (9)
Headache	4 (7)	4 (7)
Dizziness	3 (5)	1 (2)
Itching	1 (2)	3 (5)

 Only four persons missed work secondary to side effects of the prophylaxis (mean=1 day); only two physician visits for side effects occurred. No hospitalizations were reported.

## Discussion

 The findings of this study extend the data on adherence with postexposure prophylaxis and substantiate other similar surveys (11). Despite concerns about the safety of postal workers with potential exposures to *B. anthracis*, our survey demonstrates that many workers did not take adequate prophylaxis. Adherence in this population was apparently affected by a low perceived risk for anthrax and a concern about side effects. Concern about side effects was present even before postal workers started taking antibiotics; 47% of the 32 workers who never started prophylaxis cited concern about side effects as a reason. Although many workers did experience side effects, the side effects they reported were not severe. In addition, many postal workers had difficulty taking their medications as prescribed, and they missed doses of prophylaxis.

Two factors may have contributed to the low perceived risk of inhalational anthrax among postal workers. First, results from the first three efforts to collect samples at the postal facilities and the nasal swabs taken at the onset of the investigation were negative for anthrax spores. Second, postal, medical, and union leaders providing information on environmental sampling results and their interpretation at USPS town meetings tried to put the risk in the perspective as explained to them by the Department of Public Health. Overall, the data suggested a possible, but not high, risk for inhalational anthrax. Spores were likely introduced in mid-October before the New Jersey and Washington D.C. regional distribution centers that handled the contaminated Daschle and Leahy letters closed down. Use of compressed air to clean sorting machines, which might have caused aerosolization of spores, had ceased by October 23, when a general USPS advisory against it was circulated. Maximum risk of exposure to aerosolized spores likely occurred during that time. By the time the postexposure prophylaxis clinics began, 30–40 days had passed since the maximum risk period without the occurrence of any cases of inhalational anthrax in regional facility workers. In addition, the initial samples taken on November 11 and 21, with methods that readily identified spores in New Jersey and Washington, D.C., had failed to identify any spores. These factors were discussed during town meetings in an effort to reassure postal workers, while still emphasizing that a period did occur when spores were in the air, especially around the sorting machines.

 In this setting, the numbers of postal workers who accepted antibiotics could not be used as a measure for the numbers of postal workers who actually took prophylaxis. Anecdotally, many postal workers reported obtaining the antibiotics to “have on hand” in the event “I start to feel sick.” The postexposure prophylaxis survey was critical in determining the level of adherence and identifying issues affecting adherence in this population.

 The circumstances of this prophylaxis campaign, along with the small sample size and potential for recall bias associated with this survey, limit the inferences that may be drawn. For example, some misclassification of side effects as doxycycline- or ciprofloxacin-related may have accompanied the switch in medications. In addition, the study size limits any speculation as to the nature of the relationship between being men and starting prophylaxis. Larger postexposure prophylaxis surveys may identify the reason for this and other associations that were not significant in our analysis. Nonetheless, the survey provided important information on adherence to prophylaxis and reasons for nonadherence.

In the event of another bioterrorism attack, public health officials must communicate, early and effectively, the need for potentially exposed persons to initiate and continue postexposure prophylaxis. Specifically, officials should clearly communicate to at-risk persons the explanation that epidemiologic tools such as nasal swabs are poor indicators of past personal exposure and are, at best, indicators only of recent exposure. While important, reassurance must be balanced with clear explanations of risk. Of note in our study is the fact that the one group deemed to be at higher risk—those working on high-speed mail sorting machines—was found no more likely to begin or continue on prophylaxis than persons working elsewhere in the facility.

Potentially exposed persons need to be aware that side effects are to be expected, but that the vast majority of side effects will be mild. Education should center on how to recognize and minimize minor side effects while describing which side effects require immediate medical assistance. Amelioration of side effects is essential if persons are to stay on their regimens, especially if the time period is lengthy. In addition, antibiotic reminder programs such as signs in common areas or buddy systems may improve adherence to postexposure prophylaxis.

 In conclusion, if public health officials deem initiating prophylaxis programs necessary, conducting frequent follow-up surveys to measure adherence and identify obstacles to prophylaxis in a specific population will be important in identifying perception problems and maximizing the benefits of preventive therapy.
